# On the Best Mode of Applying Leeches

**Published:** 1816-04

**Authors:** J. P. O'Berne


### *For the London Medical and Physical Journal.*

### *On the best Mode of Applying Leeches;* by J. P. O'Berne, M.D.

HAVING, for some years, witnessed the very salutary effect of leeches applied in a peculiar (and, I believe, not generally known) manner, I am induced to intrude on your valuable pages; at the same time wish it to be clearly understood that I have not the smallest claim to any merit on the subject, having observed it in print many years ago.

The method is, on applying the leech, to cut off the tail, by which a *much greater* quantity of blood will be obtained than in the ordinary way.

It was particularly observable in a recent case, under a friend of mine, in this neighbourhood.

*Chillington, Devon;*

*Feb.* 26, 1816.

---

**Though the above information has appeared already in our Journal, yet we gladly offer so respectable a confirmation of what some of our readers may have overlooked. We shall be obliged to Dr. O'Berne if he can recollect in what publication he met with the remark before the London Medical and Physical Journal.—Edit.**

